# Reference Values for Fat-Soluble Vitamins in Human Milk: The Mothers, Infants and Lactation Quality (MILQ) Study

**DOI:** 10.1016/j.advnut.2025.100484

**Published:** 2025-08-26

**Authors:** Gilberto Kac, Kerry S Jones, Sarah R Meadows, Daniela Hampel, M Munirul Islam, Christian Mølgaard, Sophie E Moore, Daphna K Dror, Setareh Shahab-Ferdows, Daniela de Barros Mucci, Amanda C Figueiredo, Janet M Peerson, Lindsay H Allen, The MILQ Study Consortium, Lindsay H Allen, Sophie E Moore, Gilberto Kac, Kim F Michaelsen, Christian Mølgaard, M Munirul Islam, Maria Andersson, Setareh Shahab-Ferdows, Sophie H Christensen, Jack I Lewis, Janet M Peerson, Xiuping Tan, Daphna K Dror, Andrew M Doel, Daniela de Barros Mucci, Bruna C Schneider, Farhana Khanam, Adriana Divina de Souza Campos, Gabriela Torres Silva, Fanta Nije, Mehedi Hassan, Amanda C Figueiredo, Daniela Hampel

**Affiliations:** 1 Nutritional Epidemiology Observatory, Josué de Castro Nutrition Institute, Federal University of Rio de Janeiro, Rio de Janeiro, Brazil; 2 Nutritional Biomarker Laboratory, Medical Research Center Epidemiology Unit, University of Cambridge, Cambridge, United Kingdom; 3 USDA, ARS Western Human Nutrition Research Center, Davis, CA, United States; 4 Institute for Global Nutrition, Department of Nutrition, University of California, Davis, CA, United States; 5 Nutrition Research Division, International Centre for Diarrhoeal Disease Research, Bangladesh (icddr,b), Dhaka, Bangladesh; 6 Department of Nutrition, Exercise and Sports, Faculty of Science, University of Copenhagen, Copenhagen, Denmark; 7 Medical Research Council Unit, The Gambia at London School of Hygiene & Tropical Medicine, Fajara, The Gambia, West Africa; 8 Department of Women and Children's Health, King's College London, London, United Kingdom; 9 Department of Basic and Experimental Nutrition, Rio de Janeiro State University, Rio de Janeiro, Brazil

**Keywords:** human milk, lactation, fat-soluble vitamins, tocopherols, retinoids, vitamin D, vitamin A, vitamin E, reference values, milk volume

## Abstract

This fourth article in the series presenting reference values for nutrients in human milk describes the values for the fat-soluble vitamins A, E, and D. The Mothers, Infants and Lactation Quality (MILQ) and Early-MILQ studies collected human milk samples at multiple times during the first 8.5 mo of lactation, from 1242 well-nourished women in Bangladesh, Brazil, Denmark, and The Gambia. Vitamins A and E were measured using high-performance liquid chromatography, whereas vitamin D was measured by liquid chromatography-tandem mass spectrometry. Milk fat-soluble vitamin concentrations from the MILQ study were compared with those used by the Institute of Medicine (IOM) for setting recommendations for nutrient requirements of infants and with other selected data sets. MILQ median concentration was on par with the value used by the IOM for retinol activity equivalents except in early lactation, when they were higher. For α-tocopherol, MILQ median concentration was 76% of the IOM value. The estimate of 0.89 mg/L γ-tocopherol is novel, given that the IOM does not define a concentration in human milk. Although it is known that human milk does not meet infant requirements for vitamin D, results of the MILQ study suggest that actual median concentrations are 60%–80% of those estimated by the IOM. Total daily median intakes from 1 to 6 mo were 97%, 75%, and 6% of IOM adequate intakes for vitamin A, α-tocopherol, and vitamin D, respectively. The MILQ fat-soluble vitamin estimated percentile curves are provided to enable comparison and interpretation of data from other studies.

**Statement of Significance**

By using HPLC to measure retinol and α- and γ-tocopherol in human milk and liquid chromatography-tandem mass spectrometry to measure the low concentrations of vitamin D, reference values for these fat-soluble vitamins were developed after collecting and analyzing milk from a large number of lactating women for 8.5 mo postpartum. Although vitamin A concentrations were similar to those used by the Institute of Medicine, those for α-tocopherol and vitamin D were much lower.

## Introduction

The Mothers, Infants, and Lactation Quality (MILQ) study aimed to develop reference values (RVs) for nutrient concentrations in the milk of healthy, well-nourished mothers who were not consuming multiple micronutrient supplements. The study design and methods have been described in detail previously [1,2]. In brief, human milk samples and anthropometric, dietary, biochemical, and other data were collected from mother–infant dyads at 4 study sites (Bangladesh, Brazil, Denmark, and The Gambia). Collection occurred from 0 to 1 mo [early milk, or E-MILQ, with sample collection at 4–17 d (E1) and 18–31 d (E2)], then 3 times between 1 and 8.5 mo postpartum [1–3.49 mo (M1), 3.5–5.99 mo (M2), and 6–8.5 mo (M3)]. This article describes RVs for the fat-soluble vitamins A, E, and D. Vitamin K was not included in the analyses because milk concentration is very low, and the recommendation is to provide an intramuscular injection to infants after birth [3].

**FIGURE 1:**
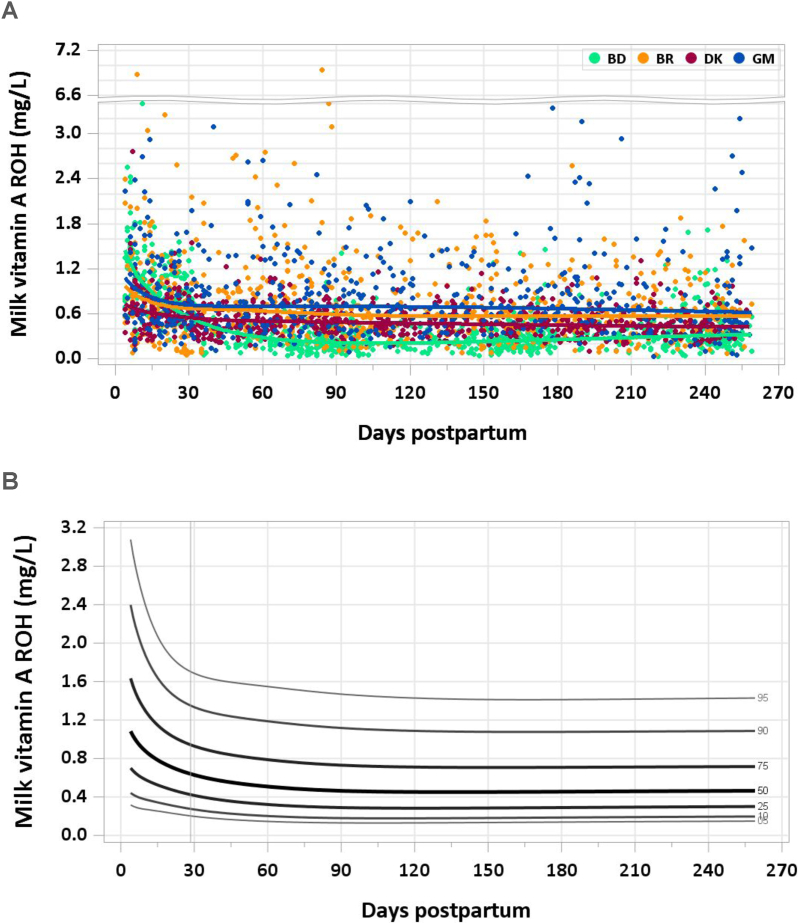
(A) Distribution of human milk retinol concentration. (B) Percentile curves for retinol concentration in human milk. BD, Bangladesh; BR, Brazil; DK, Denmark; GM, The Gambia.

**FIGURE 2:**
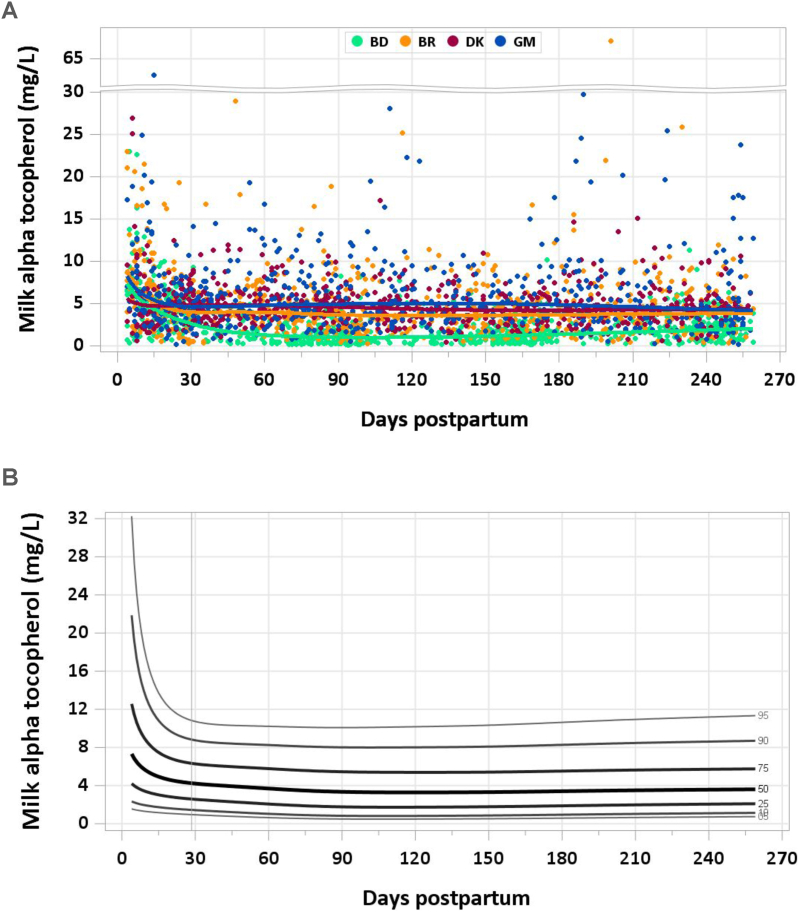
(A) Distribution of human milk α-tocopherol concentration. (B) Percentile curves for α-tocopherol concentration in human milk. BD, Bangladesh; BR, Brazil; DK, Denmark; GM, The Gambia.

**FIGURE 3:**
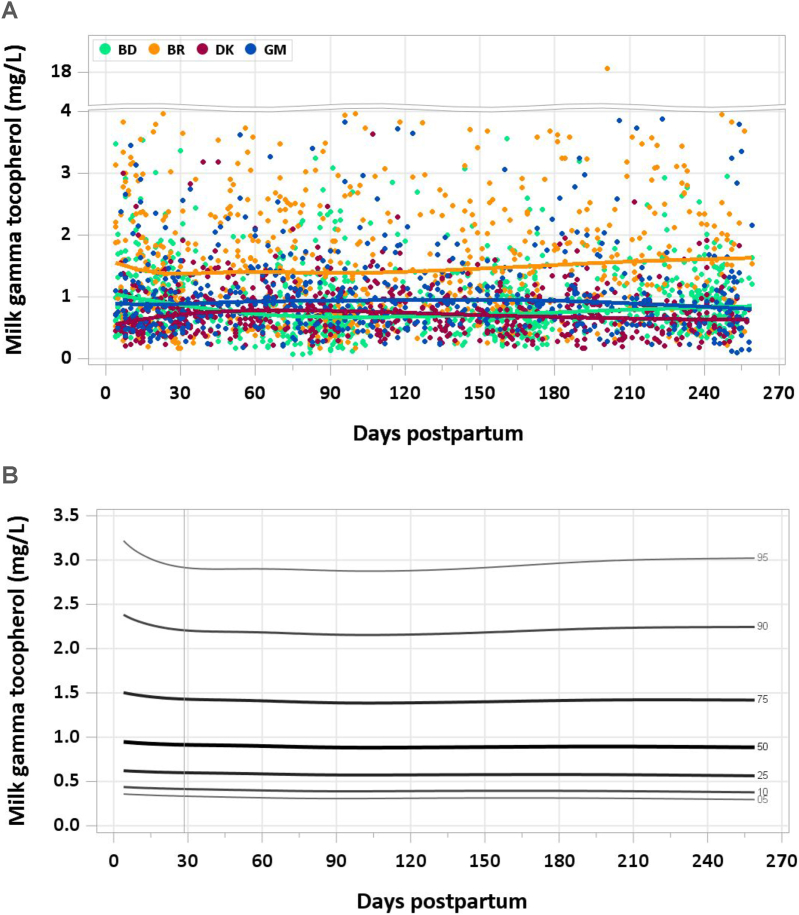
(A) Distribution of human milk γ-tocopherol concentration. (B) Percentile curves for γ-tocopherol concentration in human milk. BD, Bangladesh; BR, Brazil; DK, Denmark; GM, The Gambia.

**FIGURE 4:**
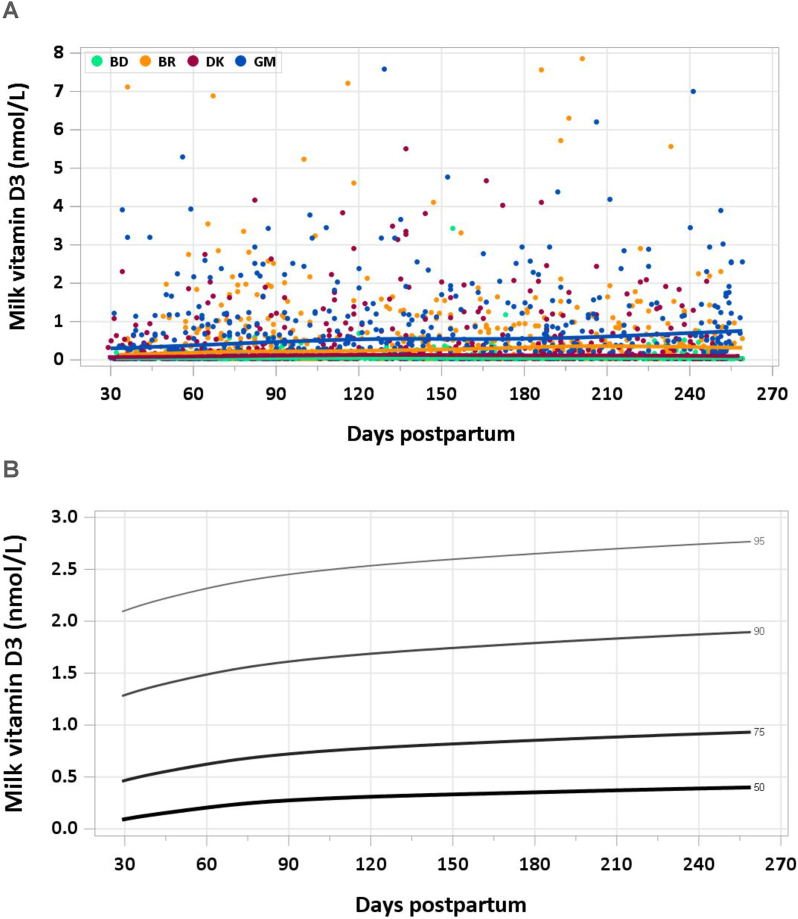
(A) Distribution of human vitamin D3 concentration. (B) Percentile curves for vitamin D3 concentration in human milk. Due to the high number of very low concentrations and reliability of estimates of the lower percentiles, percentiles below the 50th percentile are not shown. Curves constructed with data from Brazil, Denmark, and The Gambia. BD, Bangladesh; BR, Brazil; DK, Denmark; GM, The Gambia.

**FIGURE 5:**
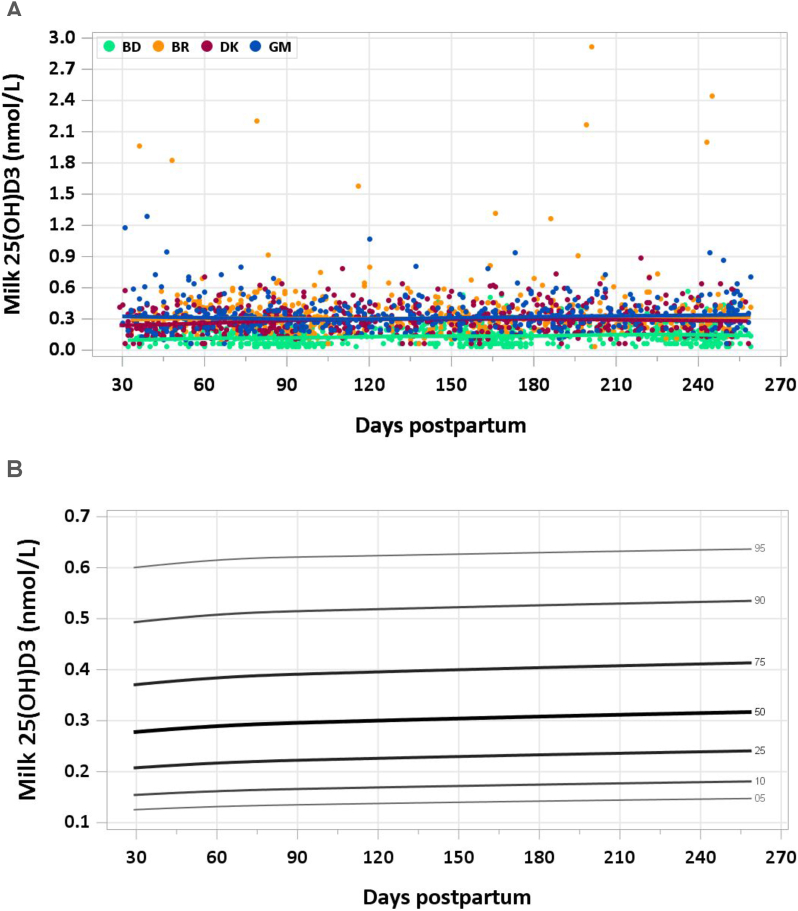
(A) Distribution of human milk 25(OH)D3 concentration. (B) Reference value curves for 25(OH)D3 concentration in human milk. Curves were constructed using data from Brazil, Denmark, and The Gambia. BD, Bangladesh; BR, Brazil; DK, Denmark; GM, The Gambia.

**FIGURE 6:**
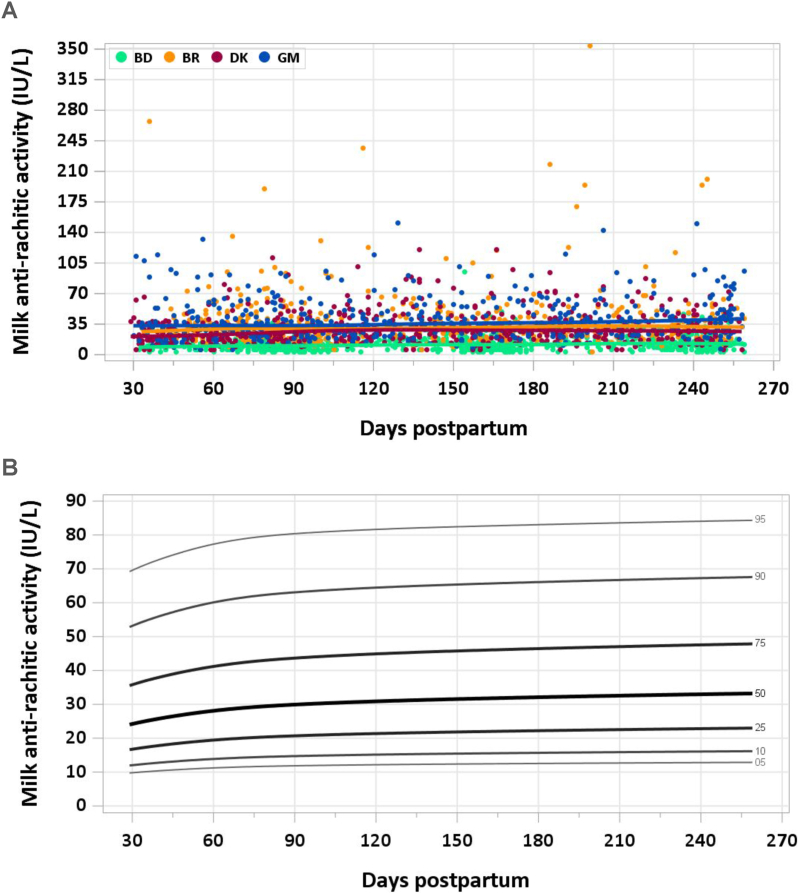
(A) Distribution of human milk antirachitic activity (ARA) concentration. (B) Reference value curves for human milk vitamin D antirachitic activity (ARA). ARA is calculated from 25(OH)D3 and vitamin D3 concentrations and assigns higher biological potency to 25(OH)D3 than vitamin D3 in a ratio of 5:1. Curves constructed with data from Brazil, Denmark, and The Gambia. BD, Bangladesh; BR, Brazil; DK, Denmark; GM, The Gambia.

This article is the fourth of a series of 7 in this supplement, which includes the introduction, RVs for macronutrients, water-soluble vitamins, minerals, and milk volumes. The supplement concludes with an article on the applications of the research. This article presents the distribution of fat-soluble vitamin concentration by site and percentile curves for fat-soluble vitamins in human milk (FIGURE 1, FIGURE 2, FIGURE 3, FIGURE 4, FIGURE 5, FIGURE 6). Also presented are comparisons between median milk fat-soluble vitamin concentrations in the MILQ study and values used by the Institute of Medicine (IOM), and comparisons between total infant daily fat-soluble vitamin intakes and existing adequate intakes (AIs) (TABLE 1, TABLE 2). Supplementary tables include estimated percentile values of nutrient concentration by month postpartum (Supplemental Table 1) as well as median total nutrient intake by study visit (1–3.49 mo, 3.5–5.99 mo, 6–8.5 mo) (Supplemental Table 2). Of note, the IOM was renamed the National Academy of Medicine (NAM) in 2015, but since dietary reference intakes for fat-soluble vitamins were published prior to the change we will refer to the institute as the IOM. A description of how the graphs and values were constructed is provided in the Introduction article in this series [2].

**TABLE 1:** Comparison of MILQ (1–6 mo) and IOM values (0–6 mo) for fat-soluble vitamin concentrations in human milk.

Nutrient	MILQ^1^ Median	MILQ IQR	IOM	MILQ as % IOM
Vitamin A (RAE, μmol/L)	1.64	(1.05–2.55)	1.70	96
ɑ-tocopherol (mg/L)	3.7	(1.8–5.6)	4.9	76
Vitamin D (ARA, IU/L)	30.6	(20.3–43.2)	40–50^2^	61–77

Abbreviations: AI, adequate intake; ARA, antirachitic activity; IOM, Institute of Medicine; MILQ, Mothers, Infants, and Lactation Quality Study; RAE, retinol activity equivalents.

1 MILQ values are pooled median concentrations from 1 to 6 mo.

2 Estimated human milk concentration, not considered to be sufficient or used to establish the AI.

**TABLE 2:** Comparison of MILQ pooled median fat-soluble vitamin intake in exclusively breastfed infants from 1 to 6 mo with IOM adequate intakes^1^.

Nutrient	MILQ	IOM AI	%AI
Vitamin A (RAE, μg/d)	389	400	97
ɑ-tocopherol (mg/d)	3	4	75
γ-tocopherol (mg/d)	0.7	N/A	—
Vitamin D (ARA, IU/d)	24.4^2^^,^^3^	400	6

Abbreviations*:* AI, adequate intake; ARA, antirachitic activity; IOM, Institute of Medicine; MILQ, Mothers, Infants, and Lactation Quality Study; N/A, not available; RAE, retinol activity equivalents.

1 AI is for 0–6 mo except vitamin D, which is for 0–12 mo.

2 Excluding Bangladesh due to a large proportion of samples with low or undetectable concentrations of vitamin D.

3 Estimated human milk concentration, not considered to be sufficient or used to establish the AI.

The graphs provided in this manuscript show the median concentration of each nutrient in milk by site and day of lactation, with individual data points in the background and the modeled 5th, 10th, 25th, 50th, 75th, 90th, and 95th percentiles by day of lactation. Estimated percentile curves were constructed using generalized additive models for location, scale, and shape (GAMLSS) [4] and the GAMLSS package (V5.4-20) using age (in days) as the only explanatory variable. Total daily intakes of each vitamin were calculated as concentration × milk volume for each mother–infant dyad at each time point. A detailed description of methods used to quantify milk volume can be found elsewhere [2]; in brief, volume was measured over 14-d periods using the stable isotope dilution dose-to-mother method except in Denmark, where before- and after-test weighing was used.

A shortage of reliable data on fat-soluble vitamins in human milk limits the ability to set nutrient requirements for infants. Some of the AIs set by the IOM for infants and lactating women are based on data on milk composition from outdated studies with few participants, inconsistent timing and methods of milk collection at various stages of lactation, lack of information about maternal diet and supplements, and inaccurate analytical methods.

Of the fat-soluble vitamins, deficiencies of vitamins A and D constitute global public health concerns. Interventions addressing vitamin A deficiency in infants and children have been in place since the 1990s [5]. The global prevalence of vitamin D deficiency and its implications for early childhood growth and development have come to light more recently. Because several forms of vitamin D were measured, a relatively larger proportion of this article is focused on this nutrient.

## Vitamin A

### Background

Vitamin A is critical for normal gene expression, reproduction, growth and physical development, erythropoiesis, immune function, and vision [6]. Because of limited transplacental transfer regardless of maternal status, infants are born with poor retinol reserves (∼5 μmol) and rely on human milk consumption to establish tissue stores, maintain growth, and develop their immune system [[7], [8], [9]]. Insufficient vitamin A intake in early lactation can predispose infants to xerophthalmia, anemia, growth retardation, depressed immune response, diarrheal disease, respiratory tract infections, and increased risk of mortality [10,11]. Vitamin A is widely distributed in animal source foods, and maternal supplementation can increase human milk concentration [12]. The contribution of pro-vitamin A carotenoids to meeting the vitamin A requirement of infants was not considered in the MILQ study because of the low concentrations and unknown bioconversion in infants [13].

### Methods

Milk retinyl esters were saponified, and the released retinol was quantified using HPLC coupled with multiwavelength detection after liquid–liquid extraction. This method offers accuracy and high sensitivity in a smaller sample (100 μL) compared with traditional HPLC methods [14].

### Results

A total of 3067 samples were analyzed (2492 MILQ, 575 E-MILQ), of which none were removed for implausible values and 306 were removed for not meeting inclusion criteria (254 MILQ, 52 E-MILQ), for a total included sample size of 2761 (2238 MILQ, 523 E-MILQ). Median milk retinol concentrations were highest in early lactation (0.886 mg/L at 4–17 d and 0.676 mg/L at 18–31 d), followed by a decrease to 0.553 mg/L at 1–2 mo. A stable concentration of 0.451–0.463 mg/L was reached by 3 mo lactation (Figure 1A and B). The pooled median total retinol intake from 1 to 6 mo was 389 μg/d.

### Comparison with published values

The IOM has set the AI for retinol activity equivalents for infants 0–6 mo at 400 μg/d based on an average milk vitamin A concentration of 0.485 mg/L and an estimated intake of 0.78 L/d. The data used to set the AI were collected from 4 studies involving 5–23 women at various points during the first 6 mo of lactation [6]. Data from the MILQ study generally support the AI of 0.485 mg/L for >3 mo lactation but suggest that a higher AI would be appropriate in early lactation. A meta-analysis published in 2022 that included 76 studies of vitamin A measured in the milk of mothers with full-term infants without recognized health concerns found mean concentrations of 0.920 mg/L in colostrum, 0.524 mg/L in transitional milk, and 0.401 mg/L in early mature milk [15]. The authors proposed that 0.40 mg/L would be an appropriate concentration to use as the basis for revising the AI because of the lower variance compared with other points in lactation. However, the median retinol concentration measured at >3 mo in the MILQ study fell at the upper end of the 95% confidence interval for the mean concentration in the meta-analysis.

## Vitamin E

### Background

Vitamin E, which comprises α, β, γ, and δ-tocopherols and α, β, γ, and δ-tocotrienols [16], is essential to the newborn to stimulate the development of the immune system and mitigate lipid peroxidation. α-tocopherol is the most bioactive form of vitamin E [17] and the form that has been measured most frequently in human milk [18]. However, γ-tocopherol is the predominant form of vitamin E in plant-derived oils and represents as much as 70% of dietary vitamin E intake in adults [19]. Due to limited transplacental transfer, circulating vitamin E concentrations are low at birth [20]. Inadequate maternal dietary intake of vitamin E does not appear to influence its concentration in human milk [19]. However, maternal supplementation with α-tocopherol or food fortification can increase human milk concentration [21].

### Methods

α- and γ-tocopherol were measured using HPLC simultaneously with vitamin A and with the same sample size.

### Results

One sample was excluded for an implausible concentration of γ-tocopherol and none for implausible concentrations of α-tocopherol. Median α-tocopherol concentration was 5.68 mg/L at 4–17 d, and decreased to 4.40 mg/L from 18 to 31 d, and to 3.92 mg/L during the second month of lactation. Median concentrations were steady at 3.30–3.60 mg/L from 2 mo onward (Figure 2A and B). In contrast, median γ-tocopherol remained steady at 0.88–0.93 mg/L throughout the study period (Figure 3A and B). The pooled median total intake from 1 to 6 mo was 3 mg/d for α-tocopherol and 0.7 mg/d for γ-tocopherol.

### Comparison with published values

The IOM has set the AI for α-tocopherol from 0 to 6 mo at 4 mg/d rounded up from 3.8 mg/d based on an average mature milk concentration of 4.9 mg/L and an average milk intake of 0.78 L/d. Data were sourced from 5 studies carried out between 1981 and 1991 that included 5–34 women and quantified α-tocopherol using HPLC [15]. The IOM recognized that α-tocopherol is higher in colostrum (average ranging from 6.8 to 23 mg/L) and transitional milk but did not recommend an AI during these periods. There are no recommended dietary intakes for γ-tocopherol. The median milk concentration of α-tocopherol in the MILQ study was 3.7 mg/L, ∼25% lower than that used by the IOM. The fall in α-tocopherol concentration from colostrum to mature milk is consistent with numerous other studies [19,[22], [23], [24], [25]]. There are fewer longitudinal data on γ-tocopherol in human milk. Although a single study in 93 samples from 48 Polish women found that milk γ-tocopherol concentration decreased throughout lactation [19], others have found stable γ-tocopherol concentrations consistent with the present results [26,27].

## Vitamin D

### Background

Vitamin D plays an important role in infant bone development and immune function. Severe deficiency is associated with rickets, whereas low vitamin D status may impact infant growth and development [28,29]. The primary source of vitamin D is cutaneous synthesis upon exposure to ultraviolet B (UVB) light; dietary intake is a secondary source. Human milk alone does not provide sufficient vitamin D to meet infant needs [30,31]. However, maternal vitamin D intake and supplementation during lactation increase the vitamin D concentration of milk [32,33]. Furthermore, the vitamin D3 content of milk was shown to increase in direct response to UVB exposure in lactating women [34]. Globally, vitamin D deficiency is present in all regions, with prevalence in neonates estimated at 29% [35].

### Methods

Vitamin D metabolites 25-hydroxyvitamin D3 (25(OH)D3), vitamin D3 (cholecalciferol), 25-hydroxyvitamin D2 (25(OH)D2), and vitamin D2 (ergocalciferol) were measured by liquid chromatography-tandem mass spectrometry using stable isotope labeled internal standards for each compound [35]. Values below the limit of quantitation (0.1 nmol/L) or limit of detection (0.05 nmol/L) were assigned values 0.1/sqrt(2) and 0.05/sqrt(2), respectively. Data are presented for 25(OH)D3 and vitamin D3 concentrations and as antirachitic activity (ARA) expressed in international units (IU/L = 25 pg/mL vitamin D3 and 5 pg/mL 25(OH)D3).

### Results

A total of 2441 samples were analyzed, of which 251 were removed for maternal or newborn factors not meeting inclusion criteria [2], and 3 vitamin D3 samples were excluded as outliers (>10 nmol/L) for a total of 2187 samples for vitamin D3 and 2190 for 25(OH)D3. Vitamin D in E-MILQ (<1 mo) was not measured due to insufficient sample volume.

#### Vitamin D3

Median vitamin D3 concentration across all sites and time points was 0.16 nmol/L. Concentrations in Bangladesh were significantly and consistently lower than in the other countries (Figure 4A). Due to the large number of low values and concentrations that were less than the limit of detection, vitamin D data for Bangladesh were not included in the calculation of the percentile curves, resulting in a sample size of 1461. Excluding Bangladesh, median country-combined vitamin D3 concentrations were 0.21, 0.32, and 0.40 nmol/L at timepoints 1–3.49, 3.5–5.99, and 6–8.5 mo, respectively. Even with the exclusion of Bangladesh, 30% of values were less than the quantification limit, resulting in a skewed dataset and, consequently, the requirement to use censored models to determine the percentile curves. A Sinh-Arcsinh (SHASH) model provided the best fit; however, due to the left-centered dataset and the resulting negative values for the 5th, 10th, and 25th percentiles, these were combined with the 50th percentile. The curves show a median vitamin D3 concentration of 0.15 nmol/L at 1–2 mo, increasing across lactation to 0.40 nmol/L at 8–8.5 mo (Figure 4B).

#### 25(OH)D3

Median 25(OH)D3 concentration across all sites and time points was 0.26 nmol/L and was less variable across time than vitamin D3. As with vitamin D3, 25(OH)D3 concentration in Bangladesh was lower than in the other countries, with an overall median concentration of 0.13 nmol/L compared with overall medians of 0.31, 0.28, and 0.32 for Brazil, Denmark, and The Gambia, respectively (Figure 5A). Bangladesh was therefore excluded from the dataset used to calculate RVs, resulting in a sample size of 1464. The percentile curves were relatively flat, with minor increases across lactation and the 50th percentile around 0.3 nmol/L (Figure 5B).

#### ARA

25(OH)D3 is assigned a higher biological activity than vitamin D3, so it has a relatively greater influence on the calculated ARA values. There were no additional exclusions to those described above for vitamin D3 and 25(OH)D3. Overall, the median ARA was 25 IU/L. In keeping with the concentration data, ARA was lowest in Bangladesh (12 IU/L) and highest in The Gambia (35 IU/L), followed by Brazil (32 IU/L) and Denmark (26 IU/L) (Figure 6A). Due to the considerably lower ARA than the other countries, Bangladesh data were not included to calculate RVs, resulting in a sample size of 1461. The modeled 50th percentile for ARA was 26 IU/L at 1–2 mo and gradually increased across lactation to 31 IU/L at 3–4 mo and 33 IU/L at 8–8.5 mo (Figure 6B). The pooled median total ARA intake from 1 to 6 mo was 24.4 IU/d.

### Comparison with published values

The low concentration of vitamin D in human milk has been recognized previously [30,31] and human milk is acknowledged by the Centers for Disease Control and Prevention in the United States to not meet the vitamin D requirements of infants [36]. The IOM states that “milk is not a meaningful source of vitamin D” [30] and estimates the concentration to be 40–50 IU/L. Represented as ARA, the median vitamin D concentration in the MILQ study was around 30 IU/L (excluding Bangladesh), 60%–75% of the IOM estimate. That vitamin D concentrations in Bangladesh are low despite year-round UVB exposure from the sun may be due to lifestyle, cultural, and environmental factors. The majority of published data, including from The Gambia [37] and Denmark [38], and using appropriate and reliable analytical methods, are consistent with the MILQ study. In Canadian women, human milk vitamin D3 and 25(OH)D3 concentrations were recently reported to be similar to those in MILQ, as 0.34 and 0.35 nmol/L, respectively [39]. The IOM set the AI for vitamin D to 400 IU/d (10 μg/d) for infants ≤12 mo based on the maintenance of serum 25(OH)D concentration >30 nmol/L; clearly, human milk provides only a small fraction of this requirement. Recommendations from most other countries are in keeping with the IOM AI [40,41].

## Discussion

The primary objective of the MILQ study was to develop RVs for nutrient concentrations in the milk of healthy mothers during the first 8.5 mo of lactation. Despite limitations in previously available data, results from the MILQ study corroborated IOM milk nutrient concentration for vitamin A, though the IOM value likely underestimates the concentration in early lactation (<3 mo). MILQ data suggest that IOM values may overestimate human milk concentrations of α-tocopherol and vitamin D expressed as ARA.

In the MILQ study, milk volume quantified by deuterium oxide dose-to-mother (or test weighing in Denmark) and milk nutrient concentration enabled the calculation of total nutrient intake by the infant at multiple time points over the first 8.5 mo of lactation. The pooled median infant intake of vitamin A from 1 to 6 mo was very close to the AI established by the IOM, whereas that for α-tocopherol was 75% of the AI. Pooled median intake of vitamin D from 1 to 6 mo in the MILQ study was only 6% of the AI, although it is well-established that human milk is an inadequate source of vitamin D for the infant [42].

The MILQ study provides the most comprehensive assessment of the fat-soluble vitamin content of human milk to date, with data from different countries and across lactation. Transfer of vitamins A and E to the infant during lactation is critical to meet needs and establish stores. Assuming that the milk of well-nourished women provides adequate nutrition for the great majority of micronutrients, the results of the MILQ study suggest that retinol needs may be higher than currently recommended early in lactation (<3 mo) whereas α-tocopherol requirements in infancy are likely lower than currently recommended. Though ɣ-tocopherol is consistently present in human milk in concentrations reflective of maternal dietary intake, more research is necessary to elucidate its bioactivity or essentiality in the infant. MILQ results confirm earlier studies showing that the vitamin D content of human milk, unlike other nutrients, is far below the concentrations necessary to independently (in the absence of infant UVB exposure) maintain adequate vitamin D status in infants. The data presented here can be used in various ways, such as to evaluate the adequacy of milk fat-soluble vitamin concentrations, assess the impact of maternal supplementation, and improve the estimates of nutrient requirements. It is hoped that where concentrations differ substantially from values used to set current AIs for infants, those will be improved and used to set estimated average requirements.

## Author contributions

The authors’ responsibilities were as follows – GK, MMI, SEM, SS-F, DH, CM, LHA: designed research; DH, SS-F, DdBM, ACF, KSJ, SRM: conducted research; DH, KSJ, SRM, JMP: analyzed data; DKD, GK, KSJ: wrote the article; LHA: had primary responsibility for final content; and all authors: read and approved the final manuscript.

## Data availability

Data described in the manuscript, code book, and analytic code will be made available on request pending application and approval.

## Funding

This article is published as part of a supplement sponsored by the Gates Foundation. Supported by the Gates Foundation (OPP1148405/INV-002300, OPP1061055) and USDA intramural funds (2023-51530-025-00D).

## Conflict of interest

The Nutritional Biomarker Laboratory (KSJ and SRM) was supported by the NIHR Cambridge Biomedical Research Centre (NIHR203312). The views expressed are those of the authors and not necessarily those of the NIHR or the UK Department of Health and Social Care.
