# Supplementary material for: Reference Values for Fat-Soluble Vitamins in Human Milk: The Mothers, Infants and Lactation Quality (MILQ) Study
Source: Adv Nutr. 2025 Aug 26;16(Suppl 1):100484. doi: 10.1016/j.advnut.2025.100484 (PMC12592235; doi:10.1016/j.advnut.2025.100484)
Supplement: multimedia component 1 [file mmc1.docx]

**Supplemental Table 1.** Monthly percentile summaries for fat soluble vitamin concentration in human milk

| Vitamin A (mg/L) | **Age** | **P05** | **P10** | **P25** | **P50** | **P75** | **P90** | **P95** |
| --- | --- | --- | --- | --- | --- | --- | --- | --- |
|  |  |  |  |  |  |  |  |  |
|  | 4-17 d | 0.27 | 0.38 | 0.59 | 0.89 | 1.31 | 1.90 | 2.42 |
|  | 18-31 d | 0.22 | 0.30 | 0.45 | 0.68 | 0.99 | 1.42 | 1.78 |
|  | 1-2 m | 0.17 | 0.23 | 0.36 | 0.55 | 0.84 | 1.24 | 1.60 |
|  | 2-3 m | 0.14 | 0.19 | 0.30 | 0.48 | 0.75 | 1.15 | 1.50 |
|  | 3-4 m | 0.13 | 0.18 | 0.29 | 0.46 | 0.72 | 1.10 | 1.45 |
|  | 4-5 m | 0.13 | 0.18 | 0.28 | 0.45 | 0.71 | 1.08 | 1.42 |
|  | 5-6 m | 0.14 | 0.18 | 0.29 | 0.45 | 0.71 | 1.08 | 1.41 |
|  | 6-7 m | 0.14 | 0.19 | 0.29 | 0.46 | 0.71 | 1.08 | 1.42 |
|  | 7-8 m | 0.15 | 0.19 | 0.30 | 0.46 | 0.71 | 1.08 | 1.42 |
|  | 8-8.5 m | 0.15 | 0.20 | 0.30 | 0.46 | 0.72 | 1.09 | 1.43 |
|  |  |  |  |  |  |  |  |  |
|  |  |  |  |  |  |  |  |  |
| Alpha tocopherol (mg/L) | **Age** | **P05** | **P10** | **P25** | **P50** | **P75** | **P90** | **P95** |
|  |  |  |  |  |  |  |  |  |
|  | 4-17 d | 1.29 | 1.94 | 3.41 | 5.68 | 8.99 | 13.94 | 18.76 |
|  | 18-31 d | 1.01 | 1.52 | 2.7 | 4.4 | 6.57 | 9.22 | 11.38 |
|  | 1-2 m | 0.77 | 1.21 | 2.29 | 3.92 | 5.98 | 8.42 | 10.34 |
|  | 2-3 m | 0.55 | 0.91 | 1.88 | 3.48 | 5.58 | 8.11 | 10.13 |
|  | 3-4 m | 0.49 | 0.82 | 1.74 | 3.3 | 5.41 | 8 | 10.12 |
|  | 4-5 m | 0.51 | 0.84 | 1.76 | 3.3 | 5.4 | 8.05 | 10.26 |
|  | 5-6 m | 0.56 | 0.91 | 1.84 | 3.37 | 5.47 | 8.19 | 10.5 |
|  | 6-7 m | 0.63 | 1 | 1.94 | 3.47 | 5.59 | 8.4 | 10.82 |
|  | 7-8 m | 0.69 | 1.07 | 2.03 | 3.55 | 5.68 | 8.56 | 11.1 |
|  | 8-8.5 m | 0.73 | 1.12 | 2.08 | 3.6 | 5.74 | 8.67 | 11.28 |
| Gamma tocopherol (mg/L) | **Age** | **P05** | **P10** | **P25** | **P50** | **P75** | **P90** | **P95** |
|  |  |  |  |  |  |  |  |  |
|  | 4-17 d | 0.35 | 0.43 | 0.61 | 0.93 | 1.47 | 2.31 | 3.09 |
|  | 18-31 d | 0.34 | 0.42 | 0.6 | 0.92 | 1.44 | 2.22 | 2.94 |
|  | 1-2 m | 0.32 | 0.41 | 0.59 | 0.91 | 1.42 | 2.19 | 2.9 |
|  | 2-3 m | 0.31 | 0.39 | 0.58 | 0.89 | 1.4 | 2.17 | 2.89 |
|  | 3-4 m | 0.31 | 0.39 | 0.57 | 0.88 | 1.39 | 2.16 | 2.88 |
|  | 4-5 m | 0.31 | 0.39 | 0.58 | 0.89 | 1.39 | 2.17 | 2.9 |
|  | 5-6 m | 0.31 | 0.39 | 0.58 | 0.89 | 1.41 | 2.2 | 2.94 |
|  | 6-7 m | 0.31 | 0.39 | 0.58 | 0.89 | 1.42 | 2.23 | 2.99 |
|  | 7-8 m | 0.3 | 0.39 | 0.57 | 0.89 | 1.42 | 2.24 | 3.01 |
|  | 8-8.5 m | 0.3 | 0.38 | 0.57 | 0.89 | 1.42 | 2.24 | 3.02 |
|  |  |  |  |  |  |  |  |  |
|  |  |  |  |  |  |  |  |  |
| Vitamin D3 (nmol/L)^1^ | **Age** | **P05** | **P10** | **P25** | **P50** | **P75** | **P90** | **P95** |
|  |  |  |  |  |  |  |  |  |
|  | 1-2 m |  |  |  | 0.151 | 0.547 | 1.392 | 2.213 |
|  | 2-3 m |  |  |  | 0.247 | 0.681 | 1.559 | 2.394 |
|  | 3-4 m |  |  |  | 0.295 | 0.755 | 1.655 | 2.5 |
|  | 4-5 m |  |  |  | 0.323 | 0.802 | 1.72 | 2.571 |
|  | 5-6 m |  |  |  | 0.344 | 0.839 | 1.771 | 2.628 |
|  | 6-7 m |  |  |  | 0.364 | 0.873 | 1.817 | 2.68 |
|  | 7-8 m |  |  | 0.051 | 0.383 | 0.904 | 1.858 | 2.726 |
|  | 8-8.5 m |  |  | 0.055 | 0.396 | 0.925 | 1.886 | 2.757 |
|  |  |  |  |  |  |  |  |  |
|  |  |  |  |  |  |  |  |  |
| 25(OH)D3 (nmol/L) | **Age** | **P05** | **P10** | **P25** | **P50** | **P75** | **P90** | **P95** |
|  |  |  |  |  |  |  |  |  |
|  | 1-2 m | 0.129 | 0.158 | 0.212 | 0.284 | 0.378 | 0.501 | 0.608 |
|  | 2-3 m | 0.134 | 0.164 | 0.22 | 0.293 | 0.388 | 0.512 | 0.619 |
|  | 3-4 m | 0.136 | 0.168 | 0.224 | 0.298 | 0.394 | 0.517 | 0.622 |
|  | 4-5 m | 0.139 | 0.171 | 0.228 | 0.302 | 0.398 | 0.521 | 0.625 |
|  | 5-6 m | 0.141 | 0.174 | 0.232 | 0.306 | 0.402 | 0.525 | 0.628 |
|  | 6-7 m | 0.143 | 0.176 | 0.235 | 0.31 | 0.406 | 0.528 | 0.631 |
|  | 7-8 m | 0.145 | 0.179 | 0.238 | 0.314 | 0.41 | 0.532 | 0.634 |
|  | 8-8.5 m | 0.147 | 0.18 | 0.24 | 0.316 | 0.413 | 0.534 | 0.636 |
|  |  |  |  |  |  |  |  |  |
|  |  |  |  |  |  |  |  |  |
| Anti-rachitic activity (IU/L) | **Age** | **P05** | **P10** | **P25** | **P50** | **P75** | **P90** | **P95** |
|  |  |  |  |  |  |  |  |  |
|  | 1-2 m | 10.6 | 13.0 | 18.2 | 26.3 | 38.7 | 56.9 | 73.7 |
|  | 2-3 m | 11.6 | 14.4 | 20.2 | 29.2 | 42.7 | 61.9 | 79.2 |
|  | 3-4 m | 12.0 | 15.0 | 21.1 | 30.5 | 44.4 | 63.9 | 81.1 |
|  | 4-5 m | 12.3 | 15.3 | 21.7 | 31.3 | 45.4 | 65.0 | 82.1 |
|  | 5-6 m | 12.5 | 15.6 | 22.1 | 31.9 | 46.2 | 65.8 | 82.8 |
|  | 6-7 m | 12.6 | 15.8 | 22.4 | 32.4 | 46.8 | 66.5 | 83.4 |
|  | 7-8 m | 12.8 | 16.0 | 22.7 | 32.8 | 47.4 | 67.1 | 83.9 |
|  | 8-8.5 m | 12.8 | 16.1 | 22.9 | 33.1 | 47.7 | 67.5 | 84.2 |

^1^For Vitamin D3, a SHASH model (sinh-arcsinh) provided the best fit; however, due to the left-centered dataset and the resulting negative values for the 5th, 10th, and 25th percentiles, these were combined with the 50th percentile.

**Supplemental Table 2**. Median infant fat soluble vitamin intake by study visit

|  | **1-3.49 mo** | **3.5-5.99 mo** | **6-8.5 mo** |
| --- | --- | --- | --- |
| **Vitamin A (μg/d)** | 386 | 398 | 308 |
| **Alpha tocopherol (mg/d)** | 2.963 | 3.054 | 2.334 |
| **Gamma tocopherol (mg/d)** | 0.71 | 0.74 | 0.596 |
| **Vitamin D (ARA**^1,2^**, IU/d)** | 23.72 | 25.89 | 23.48 |

^1^Anti-rachitic activity
^2^Excluding Bangladesh
